# High-resolution one-day probable maximum precipitation dataset across India and its future-projected changes over India

**DOI:** 10.1016/j.dib.2020.105525

**Published:** 2020-04-11

**Authors:** Subharthi Sarkar, Rajib Maity

**Affiliations:** Indian Institute of Technology, Kharagpur, India

**Keywords:** Probable maximum precipitation, Climate change, Hershfield method with modified enveloping technique

## Abstract

This article presents a high-resolution (0.25° latitude x 0.25° longitude) one-day Probable Maximum Precipitation (PMP) estimates across India based on the daily precipitation records after climate regime shift in 1970s (1971–2010). In addition to this, possible spatio-temporal changes in the PMP estimates in future towards the end of this century (2071–2100) with respect to its current values are also presented, following two possible emission scenarios (RCP4.5 and RCP8.5). These maps are developed following the research article entitled “Increase in Probable Maximum Precipitation in a Changing Climate over India”, published in Journal of Hydrology by Sarkar and Maity [1] .The recent PMP map for India is developed based on gridded daily observational records of precipitation, procured from the India Meteorological Department (IMD). Its future projection is based on bias-corrected simulated daily precipitation output from three Regional Climate Models (RCMs). Finally, the change in PMP in future with respect to the current map is calculated in terms of grid-wise percentage change and made available to the end-users. This PMP map and its future projection will serve as an important information for the design engineers and hydro-meteorologists for planning and designing various water-energy infrastructures such as, dams and other major water resources engineering applications.

Specifications Table

**Table:** 

Subject	Atmospheric Science
Specific subject area	One-day Probable Maximum Precipitation (PMP) and its future changes
Type of data	Network Common Data Form (NetCDF), Maps in Tagged Image File Format (TIFF)
How data were acquired	• Annual Maximum Daily Precipitation (AMDP): AMDP series are generated from daily precipitation data (both observed and future-simulated) and stored in 3-dimensional NetCDF format (latitude × longitude × year). • Probable Maximum Precipitation (PMP): One-day PMP is estimated across India based on observed data (1971–2010) as per Sarkar and Maity [2] and stored as descriptive map (TIFF) and 2-dimensional NetCDF format (latitude × longitude). • Future-projected changes in PMP (changePMP): To capture the future changes, multi-model averaged PMP is estimated for future (2071–2100) as per Sarkar and Maity [2] following two possible emission scenarios (RCP 4.5 and RCP 8.5), and future changes w.r.t. current PMP (1971–2010) is calculated and stored as descriptive map (TIFF) and 2-dimensional NetCDF format (latitude × longitude). • The details of the models (GCM/RCM combinations) are provided in the ‘Data Description’ section.
Data format	• AMDP series: Raw/Filtered (in the form of NetCDF file) • PMP and its future changes: Generated/Computed (in the form of NetCDF file and TIFF maps)
Parameters for data collection	The future-simulated precipitation data (2011–2100) was obtained for the domain named “WAS-44”. Then from this domain, data for Indian mainland was extracted and bias-corrected.
Description of data collection	• The gridded precipitation data set was prepared by India Meteorological Department (IMD) based on the daily rainfall records at 6955 rain gauge stations across India from 1901–2010 [3]. This data was procured from the IMD to filter out the grid wise AMDP series and to compute PMP estimates for the post-1970 period. • Simulated precipitation output from three Global Circulation Models (GCMs), downscaled by different Regional Climate models (RCMs) following two possible emission scenarios (RCP 4.5 and RCP 8.5) are downloaded from the Coordinated Regional Downscaling Experiment (CORDEX) portal: https://esgf-data.dkrz.de/search/cordex-dkrz/. These are bias-corrected and AMDP values are extracted to estimate PMP using data towards the end of the century (2071–2100). Grid wise difference in PMP estimates are obtained between current and future values.
Data source location	Country: India Observed data: Latitude: 6.50 N to 38.50 N Longitude 66.50 E to 100.0 E Future data (M1): Latitude: 6.75 N to 38.25 N Longitude: 66.75 E to 99.75 E Future data (M2 and M3): Latitude: 6.75 N to 38.25 N Longitude: 66.75 E to 99.75 E For further details about M1, M2, and M3, please see Table 1 in the related research article shown below [1].
Data accessibility	Repository name: Mendeley Data Data identification number: http://dx.doi.org/10.17632/zx99xr4wkr.1 Direct URL to data: https://data.mendeley.com/datasets/zx99xr4wkr/1
Related research article	S. Sarkar, R. Maity, Increase in Probable Maximum Precipitation in a Changing Climate over India, J. Hydrol. 124,806 (2020). doi: 10.1016/j.jhydrol.2020.124806

## Value of the data

• Recent research findings established a significant increase in PMP. Thus, the PMP estimates with the data after climate regime shift (post-1970s) must be considered for design of various water infrastructure.
• Spatio-temporal change in future-projected PMP over India provides a meaningful insight to the consequence of climate change. This information must be considered for the design of the hydraulic structures with long return periods.
• Design engineers and policy makers can use these data to revise the design of existing infrastructures due to increased risk in the context of climate change with the passage of time.
• Additionally, the future bias-corrected Annual Maximum Daily Precipitation (AMDP) series can be used extensively in different studies on future possible changes in precipitation extremes over India.

## 1 Data description

**Fig. 1:**
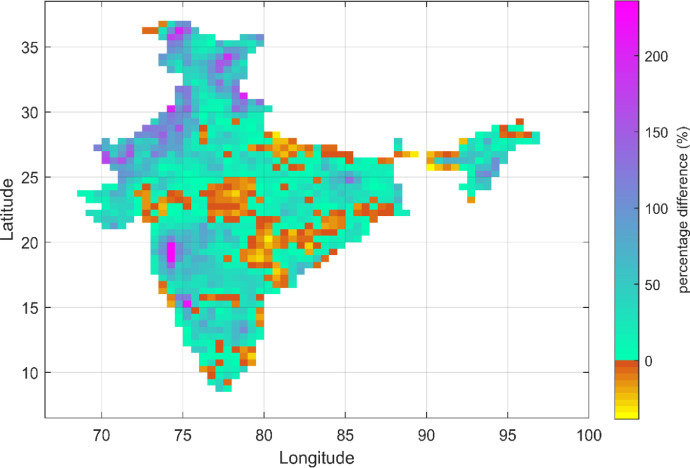
Model-averaged changes in PMP estimates in future (2071–2100) w.r.t. recent past (1971–2010) following RCP 4.5 scenario over India. The ‘cyan to purple’ portion indicates different extents of increase in PMP; whereas the ‘brown to yellow’ portion indicates different extents of reducing trend in PMP. (For interpretation of the references to color in this figure legend, the reader is referred to the web version of this article .)

**Fig. 2:**
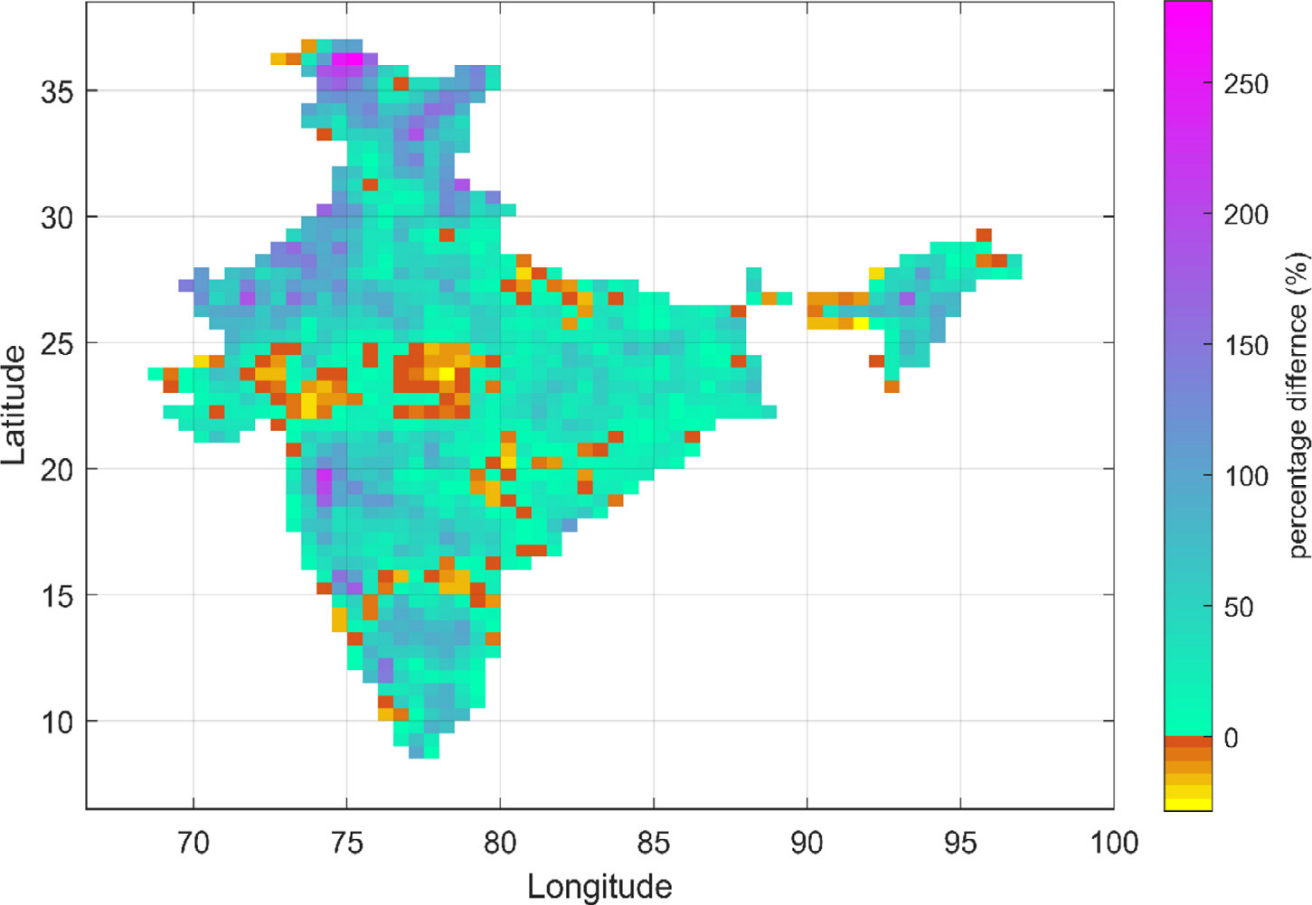
Same as Fig. 1, but following RCP 8.5 scenario.

This dataset contains the gridded AMDP series and PMP estimates over India, based on recent IMD observations (1971–2010). Additionally, it also represents multi-model AMDP series based on bias-corrected future-simulated precipitation outputs from three models (M1, M2, and M3), following two emission scenarios (RCP 4.5 and RCP 8.5) for the far-future period 2071–2100. The corresponding model-averaged (across M1, M2 and M3) changes in PMP over India w.r.t. the current values (1971–2010) are shown in Fig. 1, Fig. 2 for RCP 4.5 and RCP 8.5, respectively.

For further details, the data files are deposited at Mendeley Data, doi: http://dx.doi.org/10.17632/zx99xr4wkr.1. All data files are available in Network Common Data Form (NetCDF) format, along with descriptive maps in Tagged Image File Format (TIFF).

For comprehensive understanding, the data files are uploaded in two separate folders, as follows

• Folder name: **Based on observed data (1971–2010)**

 Filename convention: Variable_Type of data_Time period

• Folder name: **Based on future-simulated data (2071–2100)**

 Filename convention: Variable_Type of data_Model no._RCP_Time period

Variables:

  AMDP: Annual Maximum Daily Precipitation
  PMP: Probable Maximum Precipitation
  changePMP: Percentage difference in PMP in far-future period w.r.t. recent past

Type of data:

  Observed: in case of IMD observations
  Simulated: in case of bias-corrected future simulations

Model no.:

  M1: for Model-1 (GCM: MPI-ESM-LR; RCM: MPI-CSC-REMO2009)
  M2: for Model-2 (GCM: CCCma-CanESM2; RCM: SMHI-RCA4)
  M3: for Model-3 (GCM: MOHC—HadGEM2; RCM: SMHI-RCA4)
  MA: for Model-averaged estimates (averaged across M1, M2 and M3)

RCP:

  RCP45: in case of RCP 4.5
  RCP85: in case of RCP 8.5

Time period:

  Post1970: based on recent observation from 1971 to 2010
  Farfuture: based on future simulations from 2071 to 2100

To be specific, the folder ‘Based on observed data (1971–2010)**’** contains three files; two NETCDF files and one TIFF file, described as follows

1. **AMDP_observed_post1970.nc**: contains gridded (0.25° latitude  ×  0.25° longitude) Annual Maximum Daily Precipitation (AMDP) data based on recent observations from 1971–2010 over India.
2. **PMP_observed_post1970.nc**: contains gridded (0.25° latitude  ×  0.25° longitude) estimates Probable Maximum Precipitation (PMP) based on recent observations from 1971–2010 over India.
3. **PMP_observed_post1970.tif**: describes the map or spatial distribution of PMP estimates based on recent observations from 1971–2010 over India.

Similarly, the folder ‘Based on future-simulated data (2071–2100)’ contains ten files; eight NETCDF files and two TIFF files, described as follows

1. **AMDP_simulated_M1_RCP45_Farfuture.nc**: contains gridded (0.5° lat.  ×  0.5° long.) AMDP data based on future simulations from Model-1 following RCP 4.5, for far-future period (2071–2100).
2. **AMDP_simulated_M1_RCP85_Farfuture.nc**: contains gridded (0.5° lat.  ×  0.5° long.) AMDP data based on future simulations from Model-1 following RCP 8.5, for far-future period (2071–2100).
3. **AMDP_simulated_M2_RCP45_Farfuture.nc:** contains gridded (0.44° lat.  ×  0.44° long.) AMDP data based on future simulations from Model-2 following RCP 4.5, for far-future period (2071–2100).
4. **AMDP_simulated_M2_RCP85_Farfuture.nc**: contains gridded (0.44° lat.  ×  0.44° long.) AMDP data based on future simulations from Model-2 following RCP 8.5, for far-future period (2071–2100).
5. **AMDP_simulated_M3_RCP45_Farfuture.nc**: contains gridded (0.44° lat.  ×  0.44° long.) AMDP data based on future simulations from Model-3 following RCP 4.5, for far-future period (2071–2100).
6. **AMDP_simulated_M3_RCP85_Farfuture.nc**: contains gridded (0.44° lat.  ×  0.44° long.) AMDP data based on future simulations from Model-3 following RCP 8.5, for far-future period (2071–2100).
7. **changePMP_simulated MA_RCP45_Farfuture.nc**: contains gridded (0.5° lat.  ×  0.5° long.) model-averaged (across M1, M2 and M3) values of percentage changes in PMP estimates in far-future period (2071–2100) w.r.t. recent past (1971–2010), following RCP 4.5.
8. **changePMP_simulated MA_RCP45_Farfuture.tif**: describes the spatial distribution of model-averaged (across M1, M2 and M3) values of percentage changes in PMP estimates in far-future period (2071–2100) w.r.t. recent past (1971–2010), following RCP 4.5.
9. **changePMP_simulated MA_RCP85_Farfuture.nc**: contains gridded (0.5° lat.  ×  0.5° long.) model-averaged (across M1, M2 and M3) values of percentage changes in PMP estimates in far-future period (2071–2100) w.r.t. recent past (1971–2010), following RCP 8.5.
10. **changePMP_simulated MA_RCP85_Farfuture.tif**: describes the spatial distribution of model-averaged (across M1, M2 and M3) values of percentage changes in PMP estimates in far-future period (2071–2100) w.r.t. recent past (1971–2010), following RCP8.5.

## 2 Experimental design, materials, and methods

The observational dataset of PMP over the entire Indian mainland is determined based on daily gridded (0.25° latitude  ×  0.25° longitude) rainfall data, procuring from India Meteorological Department (IMD). The length of the data is 110 years, i.e., from 1901to 2010. However, in order to develop the recent PMP estimates, data for the last 40 years (1971–2010) is used.

To determine the possible changes in PMP towards the end of this century, daily simulated precipitation values for the future time period 2011–2100 (i.e., 90 years) is obtained from the Coordinated Regional Downscaling Experiment (CORDEX) portal, a reliable data archive, supported by World Climate Research Programme (WCRP). The future-simulated precipitation values are obtained for three different Regional Climate Models (RCMs), for two emission scenarios, designated by Representative Concentration Pathways (RCP 4.5 and RCP 8.5) from the CORDEX database. For further details on the data, including its driving model, source institute, data resolution for those three different model-combinations (named as M1, M2, and M3), readers can refer to Table-1 of the companion ‘Journal of Hydrology’ article [1]. To study the change in PMP towards the end of this century with respect to the current estimates (1971–2010), simulated future precipitation values are used during 2071–2100.

Future-simulated precipitation values are bias-corrected before further analysis. A recently developed copula-based bias-correction technique by Maity et al. [4] is applied, which is proven to correct the bias in both mean and extreme values, and also suitable for zero-inflated daily simulated precipitation series.

In case of both observed and future daily precipitation data, first the Annual Maximum Daily Precipitation (AMDP) time series is extracted, which is stored in the data repository. Then the method proposed by Sarkar and Maity [1,2] is applied on the AMDP series to estimate PMP. Finally, the recent PMP estimates and maps are developed and uploaded in the data repository. Similarly, multi-model averaged (average of M1, M2, and M3) PMP estimate towards the end of the 21st century are developed and changes are captured w.r.t. the current PMP estimates. The changes in terms of percentage difference are shown in the form of maps and also stored in the data-repository in NetCDF format.
